# Comparing the Diagnostic Accuracy of Magnetic Resonance Enterography and Computed Tomography Enterography in Inflammatory Bowel Diseases: A Prospective Study

**DOI:** 10.7759/cureus.97203

**Published:** 2025-11-19

**Authors:** Manish Dagar, Venkatraman Indiran, Baskar A, Saptarshi Roy, Shraddha Sharma, Piyoosh Priyadarshee

**Affiliations:** 1 Radiology, Sree Balaji Medical College and Hospital, Chennai, IND

**Keywords:** computed enterography, crohn`s disease, inflammatory bowel disease, magnetic resonance enterography, ulcerative colitis (uc)

## Abstract

Background: Inflammatory bowel diseases (IBD), comprising Crohn’s disease (CD) and ulcerative colitis (UC), are chronic relapsing inflammatory disorders requiring accurate diagnosis and periodic monitoring. Cross-sectional imaging modalities such as magnetic resonance enterography (MRE) and computed tomography enterography (CTE) have become pivotal in evaluating bowel wall and extraintestinal involvement. This study aimed to compare the diagnostic efficacy and feature agreement between MRE and CTE in assessing key mural and extramural characteristics of IBD.

Methods: A comparative cross-sectional study was conducted on patients diagnosed with IBD who underwent both MRE and CTE. Imaging findings were independently reviewed to assess diagnostic agreement on parameters including mural thickening, mural stratification, skip lesions, luminal stenosis, and creeping fat sign. Statistical analyses were performed using chi-square tests (χ²) and independent t-tests to evaluate agreement, discrepancies, and demographic correlations.

Results: The mean age of participants was 43.40 ± 13.38 years (range: 20-63 years), with 19 (54.3%) patients diagnosed with CD and 16 (45.7%) with UC. No significant difference was observed in age between agreement and discrepancy groups (t = -0.05, p = 0.9611). Overall, MRE showed higher agreement with clinical diagnosis for mural stratification (p<0.05) and creeping fat (p<0.01) than CTE. Feature-wise comparison revealed agreement rates of 68.97% for mural thickening (χ² = 0.18, p = 0.6756), 74.19% for mural stratification (χ² = 0.0, p = 1.0), 66.67% for skip lesions (χ² = 0.0, p = 0.9901), 63.64% for luminal stenosis (χ² = 0.19, p = 0.6589), and 66.67% for creeping fat (χ² = 0.27, p = 0.6044).

Conclusion: MRE and CTE demonstrated comparable diagnostic agreement in evaluating IBD features, with MRE offering superior tissue characterization and radiation-free imaging, while CTE provided faster acquisition and higher spatial resolution. Despite moderate agreement levels, both modalities remain complementary in clinical practice. MRE should be preferred for longitudinal follow-up, particularly in younger patients, given its safety profile and diagnostic comprehensiveness.

## Introduction

Inflammatory bowel disease (IBD) represents a group of chronic, relapsing inflammatory disorders of the gastrointestinal (GI) tract, primarily comprising Crohn’s disease (CD) and ulcerative colitis (UC). These conditions are characterized by alternating periods of exacerbation and remission, leading to significant morbidity and a substantial impact on patients’ quality of life [1]. The pathogenesis of IBD is multifactorial, involving complex interactions among genetic predisposition, environmental triggers, gut microbiota alterations, and dysregulated immune responses [2]. This aberrant immune activation results in persistent intestinal inflammation, mucosal injury, and progressive structural damage, often culminating in complications such as strictures, fistulas, and abscesses [2].

Over the past few decades, the global burden of IBD has increased markedly. Since 1990, IBD has been increasing in newly industrialized countries. For example, in Brazil, CD rose by an average of 11.1% per year and UC by 14.9%, while in Taiwan, CD increased by 4.0% per year and UC by 4.8%. Although once considered a disease predominantly affecting Western populations, its prevalence has risen in newly industrialized and developing nations, including those in Asia, paralleling trends of urbanization, dietary westernization, and lifestyle transitions [3]. This epidemiological shift underscores the pressing need for robust diagnostic and monitoring modalities, particularly in regions where healthcare resources are limited and the long-term management of chronic diseases poses major challenges [3].

CD and UC, while sharing overlapping clinical and pathological features, differ significantly in their anatomical distribution and pattern of inflammation. CD may involve any part of the GI tract from the mouth to the anus, most commonly affecting the terminal ileum and proximal colon. It is characterized by discontinuous (“skip”) lesions and transmural inflammation, which penetrates the entire bowel wall, often leading to complications such as strictures, fistulas, and abscess formation [4]. Conversely, UC is confined to the colon and rectum, with inflammation limited to the mucosal and submucosal layers. It typically presents as continuous inflammation starting from the rectum and extending proximally [4]. Despite these differences, both diseases necessitate accurate diagnosis, continuous disease monitoring, and individualized management to prevent complications and improve patient outcomes [5].

The management of IBD is inherently multidisciplinary, involving collaboration among gastroenterologists, radiologists, surgeons, and pathologists [6]. Radiological imaging has become an indispensable component of this approach, complementing clinical, endoscopic, and laboratory assessments in both diagnosis and follow-up. Imaging is particularly crucial in CD, given its frequent involvement of the small bowel, a region that is challenging to evaluate endoscopically [6]. The small intestine’s considerable length, mobility, and deep anatomical position make it difficult to assess using traditional endoscopy, thereby necessitating advanced cross-sectional imaging techniques [7].

Historically, small bowel evaluation relied on conventional radiographic techniques such as the barium meal follow-through. This method, which involves ingestion of contrast followed by serial X-rays, provides visualization of the bowel lumen but offers limited information regarding the bowel wall and adjacent structures [7]. Its inability to detect subtle inflammatory changes, transmural disease, or extraintestinal complications rendered it suboptimal for comprehensive IBD assessment [7]. Consequently, reliance on such conventional modalities has declined, giving way to modern cross-sectional imaging techniques, notably computed tomography (CT) and magnetic resonance imaging (MRI) [8].

Among these, CT enterography (CTE) has emerged as a highly effective and widely accessible tool for evaluating small bowel involvement in IBD. By combining multidetector CT imaging with oral and intravenous contrast agents, CTE enables detailed assessment of the bowel wall, lumen, and mesentery. It excels in detecting mural thickening, mural stratification, luminal narrowing, skip lesions, and extraintestinal complications such as abscesses and fistulas [8,9]. The major strengths of CTE include its high spatial resolution, rapid acquisition time, and broad availability, making it invaluable in emergency and acute care settings [9]. However, its reliance on ionizing radiation remains a critical limitation, especially in younger patients and those requiring repeated imaging for disease monitoring. Cumulative radiation exposure has been associated with increased long-term risks, including malignancy, underscoring the need for safer alternatives [10].

In recent years, MR enterography (MRE) has gained prominence as a radiation-free imaging modality offering comparable diagnostic performance to CTE [11]. Utilizing magnetic fields and radiofrequency waves, MRE provides superior soft-tissue contrast and multiplanar imaging capabilities, allowing comprehensive evaluation of the bowel wall and surrounding structures [11]. It can also assess functional parameters, including bowel motility and inflammatory activity, through contrast enhancement and diffusion-weighted imaging sequences [12]. MRE has demonstrated high sensitivity and specificity in detecting active inflammation in CD, identifying characteristic features such as mural thickening, stratification, luminal stenosis, skip lesions, and creeping fat, a distinctive radiologic hallmark of chronic transmural inflammation [12]. This study aims to compare the effectiveness of MRE and CTE in detecting IBD by assessing both wall-related (mural) and outside-the-wall (extra-mural) features, including thickening, layer changes, skip lesions, narrowing of the bowel, and the presence of creeping fat.

## Materials and methods

Study design

This study was designed as a prospective, cross-sectional, comparative diagnostic imaging investigation conducted in the Department of Radiodiagnosis and Imaging at Sree Balaji Medical College and Hospital, Chennai, India. The study period extended from July 2023 to June 2025. The primary objective was to compare the diagnostic performance of MRE and CTE in the evaluation of patients with clinically suspected IBD. Particular emphasis was placed on the identification and characterization of both mural and extramural features associated with CD and UC. By using both imaging modalities in the same cohort, the study sought to assess diagnostic agreement, sensitivity, and the relative advantages of each technique.

Study population and sampling technique

A total of 35 adult patients were enrolled using a convenience sampling technique. Eligible participants were referred from the Departments of Medicine, Surgery, and Gastroenterology based on a strong clinical suspicion of IBD. Patients exhibiting typical clinical manifestations, such as chronic abdominal pain, diarrhea, malabsorption, unexplained weight loss, and anemia, were considered for inclusion. In addition to clinical presentation, biochemical and endoscopic findings suggestive of CD or UC were used to support eligibility. All patients were briefed about the study objectives and procedures, and written informed consent was obtained before participation.

Sample size calculation

The sample size was calculated to compare the diagnostic yield between CTE and MRE in detecting intestinal lesions. Assuming a 95% confidence level (Zα/2 = 1.96), 80% power (Zβ = 0.84), an expected detection proportion of 0.80 for CTE and 0.95 for MRE, and a clinically significant difference of 15% (0.15), the required sample size per group was 73 participants per group.

The study aimed to achieve adequate statistical power to detect differences between the two imaging modalities in terms of diagnostic accuracy. Although the final sample size was 35, the calculation was guided by standard diagnostic accuracy study formulas, considering parameters such as expected sensitivity, prevalence of disease, allowable error, and confidence level. The sample size was balanced against practical constraints, including patient availability and imaging resources, to ensure feasibility within the study period.

Inclusion and exclusion criteria

Participants aged above 18 years who had a clinical suspicion or provisional diagnosis of IBD and were willing to undergo both MRE and CTE were included. Written informed consent was a prerequisite for participation. Patients with contraindications to MRI, such as those with cardiac pacemakers, cochlear implants, metallic prostheses, or severe claustrophobia, were excluded. Additional exclusion criteria included a known allergy to iodinated contrast agents, renal insufficiency with estimated glomerular filtration rate (eGFR) below 30 mL/min/1.73 m², pregnancy or lactation, and unstable patients requiring emergency surgical intervention. These exclusion parameters were applied to ensure patient safety and data reliability.

Ethical considerations

Ethical approval was obtained from the Institutional Human Ethics Committee (IEC) of the institution before the commencement of the study (approval number: 002/SBMC/IHEC/2023/1969). Participants were informed in detail about the study’s purpose, methodology, potential risks, and benefits. Participation was entirely voluntary, and confidentiality was strictly maintained. Each participant was assigned a unique study code to preserve anonymity. The study was conducted in accordance with the ethical principles outlined in the Declaration of Helsinki and adhered to institutional policies on biomedical research involving human subjects.

Patient preparation

To maintain uniformity between the two imaging modalities, standardized patient preparation protocols were implemented. All patients were instructed to fast overnight prior to imaging to minimize bowel content and motion artifacts. Adequate hydration was ensured both before and after the procedures to facilitate contrast clearance and reduce nephrotoxicity. The procedure was explained clearly to each patient to alleviate anxiety and ensure compliance. For both MRE and CTE, oral contrast administration using a polyethylene glycol (PEG-3350) solution was standardized to achieve optimal small bowel distension and consistent luminal visualization.

CTE protocol

CTE was performed using a 32-slice SIEMENS SOMATOM go CT scanner (Siemens Healthineers, Erlangen, Germany). Each patient ingested approximately 1.5 liters of PEG-3350 solution dissolved in water over 45-60 minutes before scanning. This ensured uniform small bowel distension, which is critical for accurate evaluation of mural and luminal abnormalities. Following oral preparation, 80 mL of non-ionic iodinated contrast (Iohexol 350 mg/mL) was administered intravenously using a pressure injector at a rate of 3-4 mL per second. Scanning was carried out from the dome of the diaphragm to the pubic symphysis, capturing both arterial and venous phases. Images were reconstructed in axial and coronal planes with a slice thickness of 3 mm. Radiation dose optimization techniques, including automatic exposure control and dose modulation, were employed to minimize radiation exposure, particularly for younger patients or those requiring repeated follow-up imaging. The resulting images provided a high-resolution visualization of bowel wall thickening, mural stratification, skip lesions, and extraintestinal complications such as abscesses and fistulas.

MRE protocol

MRE was conducted on a SIGNA PIONEER 3.0 Tesla MRI system (GE Healthcare, Chicago, IL) equipped with a phased-array body coil. The oral contrast preparation was identical to that used in CTE, with 1.5 liters of PEG solution administered over 45-60 minutes to achieve consistent bowel distension. Immediately before imaging, 20 mg of intravenous hyoscine butylbromide was given to reduce bowel peristalsis and motion artifacts. The imaging protocol included multiple sequences to capture structural and functional bowel details. These comprised axial and coronal T2-weighted fast spin echo images, T2-weighted fat-suppressed short tau inversion recovery (STIR) sequences, and T1-weighted pre-contrast gradient echo images in both axial and coronal planes. Balanced steady-state free precession (SSFP) sequences were obtained with and without fat suppression to enhance visualization of bowel loops and mesentery. Dynamic post-contrast T1-weighted images were acquired following the intravenous administration of gadolinium-based contrast agents (0.1 mmol/kg body weight). In cases with suspected perianal disease, additional high-resolution axial and coronal images through the pelvis were obtained. All images were reconstructed using multiplanar reformats and maximum intensity projections for optimal evaluation.

Imaging evaluation criteria

The imaging studies were interpreted independently by three experienced radiologists, with each having three to five years of expertise in gastrointestinal imaging. Each scan was assessed for specific parameters, including mural thickening (defined as bowel wall thickness greater than 3 mm), mural stratification (enhanced mucosa with a hypo-enhancing submucosa indicative of edema), skip lesions (discontinuous involvement of bowel segments separated by normal bowel), luminal stenosis and proximal dilatation, and the presence of the creeping fat sign (mesenteric fat proliferation adjacent to affected bowel segments). Each parameter was recorded in a binary manner (present or absent). Findings from CTE and MRE were compared to evaluate diagnostic agreement and the relative sensitivity of each modality.

Data collection and management

All patient information, including demographic data, clinical details, laboratory results, and imaging findings, was systematically recorded in a predesigned data sheet using Microsoft Excel (Microsoft Corp, Redmond, WA). Data were anonymized using unique study codes to ensure confidentiality. The dataset was maintained securely with restricted access to authorized research personnel only. Internal consistency checks were performed regularly to ensure accuracy and completeness.

Statistical analysis

Data were analyzed using IBM SPSS Statistics software, version 20.0 (IBM Corp., Armonk, NY). Descriptive statistics, including mean, median, standard deviation, and range, were used to summarize continuous variables such as age. The independent samples t-test was used to compare mean age differences between groups with concordant and discordant imaging findings. Categorical variables, such as the presence or absence of imaging features and the type of diagnosis (CD or UC), were analyzed using the chi-square (χ²) test of independence. A p-value of less than 0.05 was considered statistically significant. Statistical analyses were designed to assess the strength of association between imaging findings and diagnostic agreement across modalities.

## Results

A total of 35 patients were included in the study. The mean age was approximately 43.4 years (range: 20-63 years), with a standard deviation of ~11.6 years. Among the participants, 19 (54.3%) were diagnosed with CD and 16 (45.7%) with UC.

In this cohort of 35 patients (mean age 43.4 ± 11.6 years), MRE demonstrated higher detection rates for most imaging features compared to CTE. Mural thickening was observed in 30 (85.7%) patients on MRE and 28 (80.0%) on CTE, while mural stratification was identified in 26 (73.6%) and 20 (57.8%) patients, respectively. Skip lesions were detected in 24 (68.4%) cases on MRE versus 22 (63.1%) on CTE, and creeping fat was noted in 25 (71.0%) and 17 (48.0%) cases, respectively. The detection of luminal stenosis was nearly similar between MRE (23 (65.7%)) and CTE (24 (68.4%)) of key inflammatory and transmural changes in bowel pathology, reinforcing its diagnostic advantage over CTE in assessing inflammatory bowel disease (Table 1).

**Table 1:** Detection Rates of Imaging Features on MRE and CTE Among the Study Population (n = 35) MRE: Magnetic Resonance Enterography; CTE: Computed Tomography Enterography

Imaging Feature	MRE: n (%) (95% CI)	CTE: n (%) (95% CI)
Mural Thickening	30 (85.7%) (73.3–98.1)	28 (80.0%) (65.7–94.3)
Mural Stratification	26 (73.6%) (57.9–89.3)	20 (57.8%) (40.8–74.8)
Skip Lesions	24 (68.4%) (51.8–85.0)	22 (63.1%) (46.0–80.2)
Luminal Stenosis	23 (65.7%) (48.8–82.6)	24 (68.4%) (51.8–85.0)
Creeping Fat	25 (71.0%) (54.8–87.2)	17 (48.0%) (30.2–65.8)

**Figure 1:**
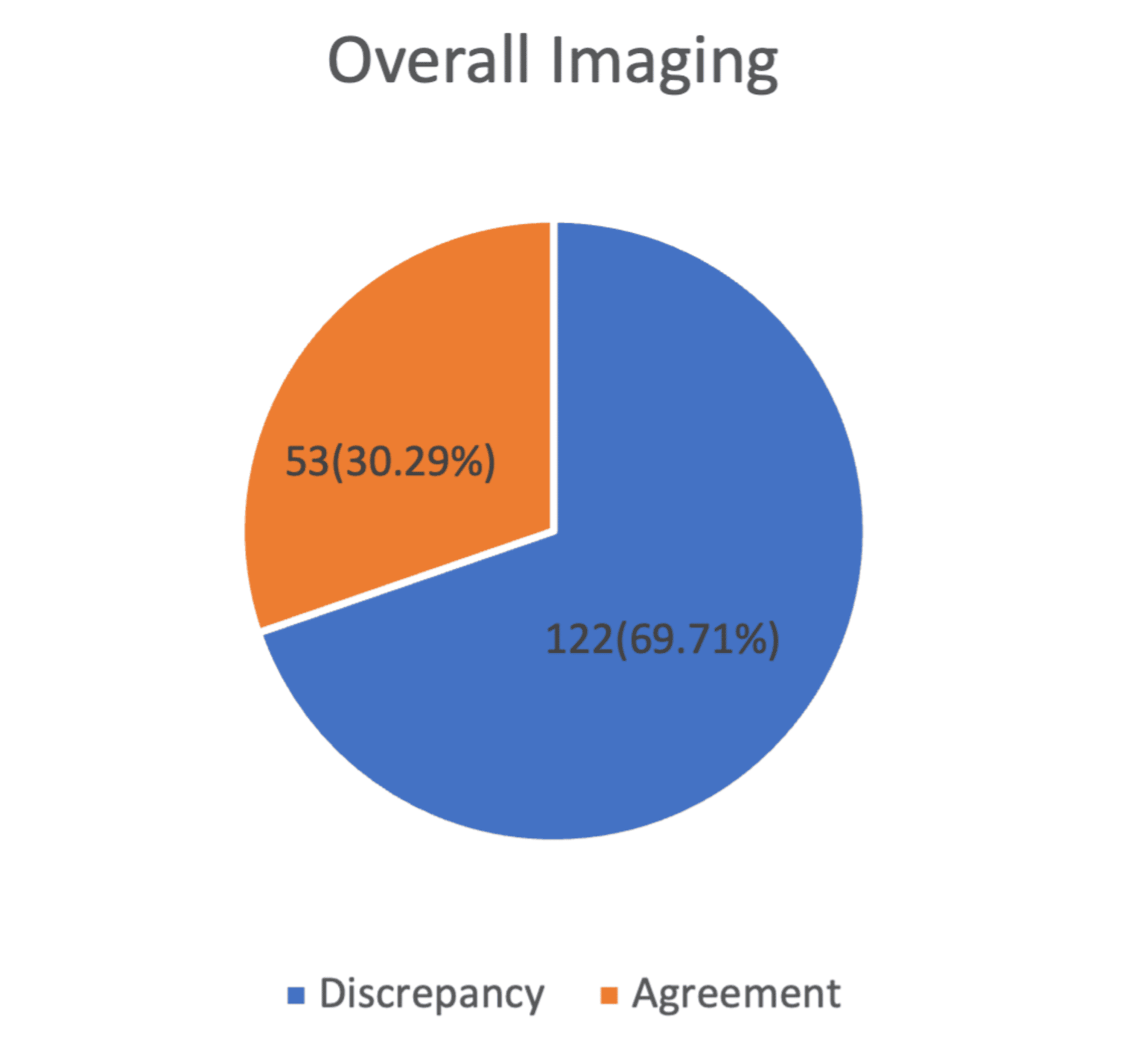
Overall Imaging Agreement vs. Discrepancy The Data Are Reprsented as N(%) in the Figure.

Out of a total of 175 feature comparisons, 122 cases (69.7%) demonstrated concordance between the assessed parameters, indicating a high level of consistency across measurements. However, 53 cases (30.3%) showed discrepancies, suggesting variability in certain features that may reflect inter-individual differences or measurement limitations. Overall, the agreement rate underscores the reliability of the assessment while highlighting areas requiring cautious interpretation (Figure 1).

The comparison of imaging features across the cohort revealed varying degrees of agreement and discrepancy. The mean age was similar between the agreement (43.57 ± 13.94 years) and discrepancy groups (43.40 ± 13.38 years), with no significant difference (t = 0.04, p = 0.972). For disease-specific features, 16 patients (45.7%) with CD and 14 patients (46.7%) with UC showed agreement, while discrepancies were observed in 19 (54.3%) and 16 (53.3%) patients, respectively. Imaging signs such as mural thickening and mural stratification had equal or slightly lower agreement rates, with three (50.0%) and 11 (44.0%) cases concordant and three (50.0%) and 14 (56.0%) cases discordant. Skip lesions were in agreement in 15 patients (44.1%) and discrepant in 19 (55.9%), while luminal stenosis and the creeping fat sign showed agreement in 18 (45.0%) and 12 (42.9%) patients and discrepancy in 22 (55.0%) and 16 (57.1%) patients, respectively. None of the features demonstrated statistically significant differences (p > 0.05), reflecting a moderate consistency in imaging evaluation across the assessed cohort (Table 2).

**Table 2:** Associations of Imaging Agreement and Features in Inflammatory Bowel Disease * Chi-Square/Fisher’s Exact Test; Independent Samples T-test; P-value < 0.05 is Statistically Significant.

Imaging Feature	Agreement n (%)	Discrepancy n (%)	Chi-square (χ²)/t-value*	p-value
Age (Mean+SD)	43.57 ± 13.94	43.40 ± 13.38	0.04	0.972
Crohn's Disease	16 (45.71%)	19 (54.29%)	0.11	0.802
Ulcerative Colitis	14 (46.67%)	16 (53.33%)	0.14	0.798
Mural Thickening	3 (50.0)	3 (50.0)	0.18	0.676
Mural Stratification	11 (44.0)	14 (56.0)	0.14	0.702
Skip Lesions	15 (44.1)	19 (55.9)	0.11	0.741
Luminal Stenosis	18 (45.0)	22 (55.0)	0.10	0.754
Creeping Fat Sign	12 (42.9)	16 (57.1)	0.15	0.698

**Figure 2:**
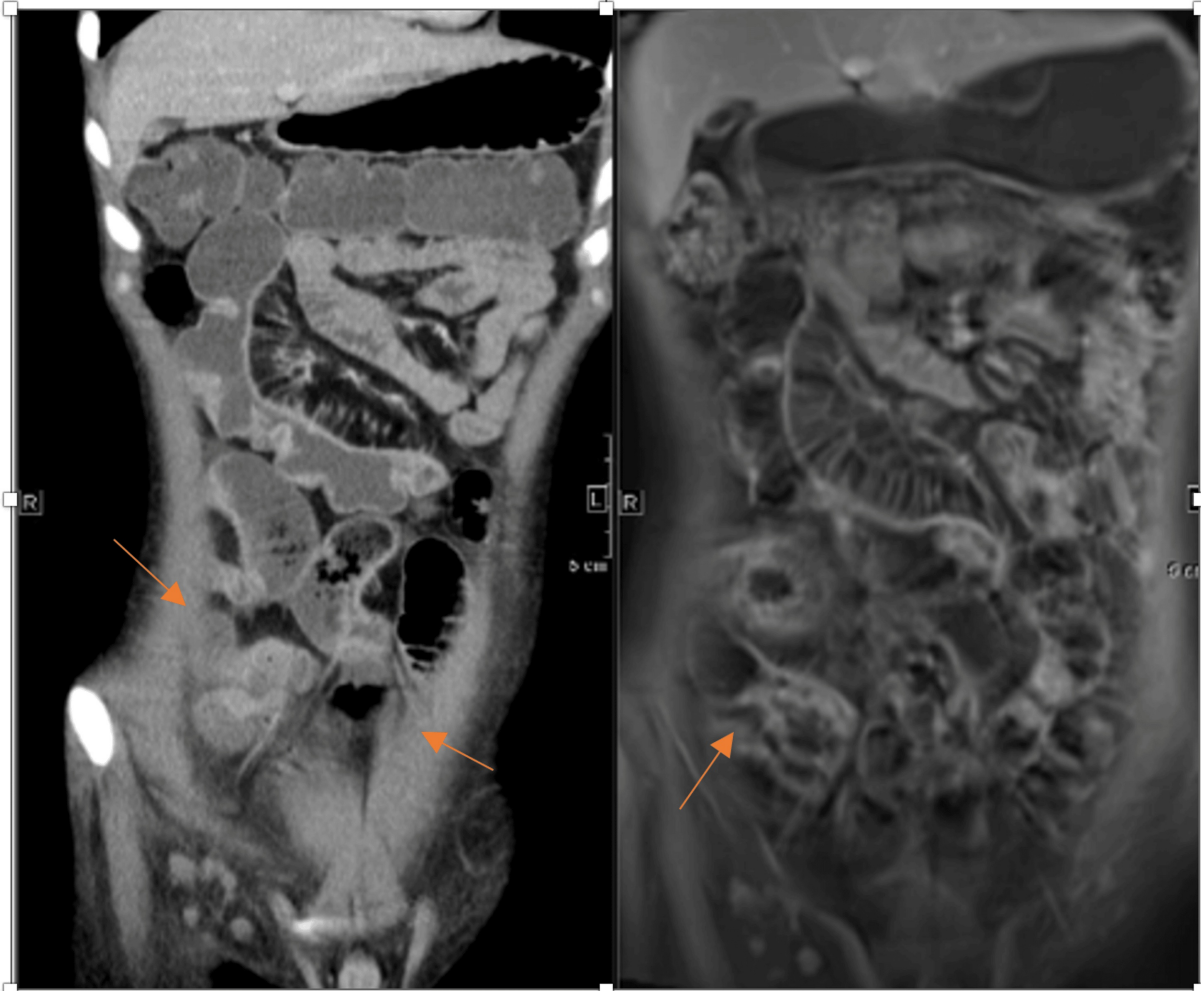
Creeping Fat With Prominent Vasa Recta on a) CTE Coronal Image and B) T1 Post-contrast Coronal Image (Arrow) CTE: Computed Tomography Enterography

The coronal CTE and post-contrast T1 images demonstrated classic “creeping fat,” characterized by hypertrophy of mesenteric fat encasing the affected bowel loops, with numerous engorged and elongated vasa recta radiating toward the bowel wall, producing the typical “comb sign.” These findings reflect active mesenteric inflammation and vascular proliferation, commonly seen in CD, indicating transmural involvement and ongoing inflammatory activity (Figure 2).

**Figure 3:**
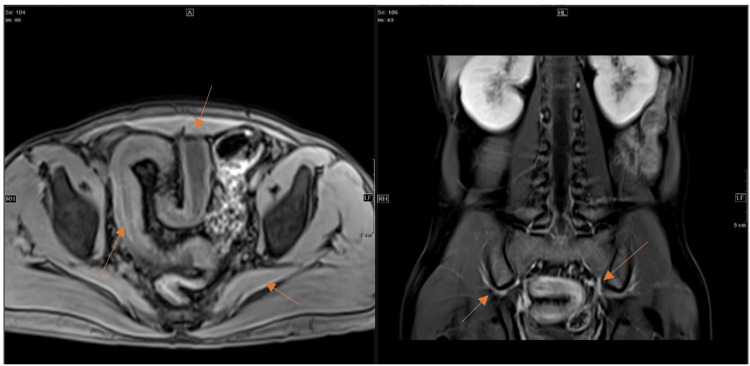
T1 Post-contrast Axial and Coronal MRE Images Showing Long-Segment Mural Thickening With Stratification in the Sigmoid Colon (Arrows) MRE: Magnetic Resonance Enterography

Post-contrast axial and coronal MRE images showed a long segment of mural thickening involving the sigmoid colon with a preserved layered (stratified) enhancement pattern: hyperenhancing mucosa, hypointense submucosa, and enhancing serosa (“target sign”). The stratification suggested active inflammatory disease with submucosal edema and hyperemia, consistent with an acute flare of Crohn’s colitis rather than fibrotic or quiescent disease (Figure 3).

**Figure 4:**
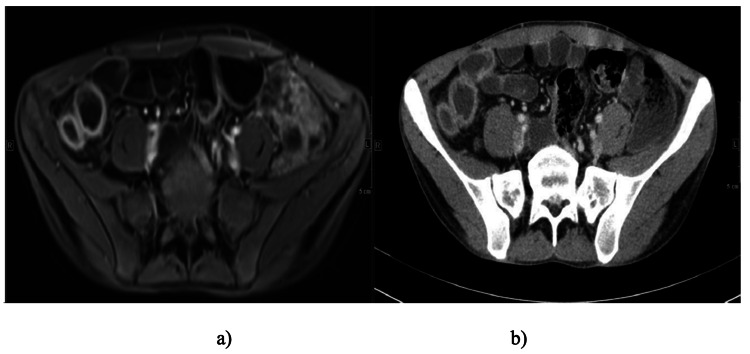
Mural Thickening Without Stratification in the Sigmoid Colon in a) T1 Post-contrast Axial and B) Coronal MRE MRE: Magnetic Resonance Enterography

Figure 4 demonstrates a homogeneous, unstratified mural thickening of the bowel wall, lacking the layered contrast enhancement seen in acute inflammation. The absence of stratification suggested chronic fibrotic change or quiescent disease, reflecting transmural fibrosis and loss of normal mural architecture. These imaging features are typical of long-standing CD with predominantly fibrostenotic transformation rather than active inflammation.

## Discussion

IBDs, encompassing CD and UC, are chronic, relapsing-remitting disorders characterized by inflammation of the gastrointestinal tract. Both diseases impose a significant physical and psychological burden on affected individuals due to their fluctuating nature, long-term course, and the potential for severe complications, such as strictures, fistulas, and abscesses. Consequently, they require continuous medical surveillance and periodic imaging to assess disease activity, detect complications, and evaluate treatment response [13,14]. The complexity of IBD lies in its variable presentation and multifactorial pathogenesis, involving genetic, environmental, microbial, and immune-mediated mechanisms. Accurate diagnosis and longitudinal monitoring are therefore crucial, and imaging plays a pivotal role in this multidisciplinary management. Among available modalities, MRE and CTE have become the mainstay techniques for small bowel evaluation, especially in CD, where inflammation frequently extends through the entire bowel wall and may involve discontinuous intestinal segments [15].

CTE combines rapid image acquisition with high spatial resolution, providing detailed visualization of the intestinal wall and surrounding mesentery. It utilizes neutral oral contrast agents alongside intravenous iodinated contrast to delineate both luminal and extramural disease features. Its major strength lies in the ability to detect acute complications such as perforation, obstruction, and abscesses, which demand prompt surgical or medical intervention. However, a key drawback of CTE is its reliance on ionizing radiation. Repeated exposure, especially in younger patients requiring serial follow-up, raises concerns regarding cumulative radiation dose and long-term carcinogenic risk [10].

By contrast, MRE offers an equally comprehensive evaluation without radiation exposure. It provides superior soft-tissue contrast and functional assessment, allowing visualization of bowel motility, vascularity, and wall enhancement patterns. MRE employs oral contrast for bowel distension and gadolinium-based intravenous agents for highlighting mucosal enhancement, combined with sequences such as T2-weighted and diffusion-weighted imaging. These allow differentiation between active inflammation (edematous, hyperenhancing bowel wall) and chronic fibrotic disease (thickened, hypoenhancing wall with minimal diffusion restriction). Despite a relatively lower spatial resolution and longer acquisition time compared to CTE, MRE’s ability to avoid radiation and characterize disease activity at the tissue level makes it invaluable, particularly for children, adolescents, and young adults [16].

Previous comparative studies have shown that both CTE and MRE possess high sensitivity and specificity in detecting active IBD [7,10-13]. However, MRE provides better reproducibility and inter-observer agreement in identifying disease activity and severity. While the cost, scan duration, and limited availability of MRE can restrict its use, CTE remains a dependable alternative in emergency scenarios or in institutions with limited MRI access [17]. Thus, selecting between the two modalities depends on patient age, urgency, disease activity, and resource settings, highlighting the importance of an individualized imaging approach [18]. In the present study, the mean age of participants was 43.40 ± 13.38 years (range: 20-63 years). Statistical comparison using the independent t-test revealed no significant difference between the imaging agreement and discrepancy groups (t = -0.05, p = 0.9611). This indicates that age did not influence diagnostic concordance between the two modalities. Similarly, diagnosis-based analysis revealed no significant association between imaging agreement and disease type, with CD and UC showing similar levels of consistency. These findings emphasize that both CTE and MRE can be applied across the IBD spectrum, regardless of disease subtype.

Feature-based comparison revealed further nuances in diagnostic agreement. For mural thickening, concordance between CTE and MRE was observed in 68.97% of cases (χ² = 0.18, p = 0.6756). Mural thickening, a hallmark of transmural inflammation, is typically well visualized on both modalities. CTE’s spatial resolution allows precise delineation of bowel wall thickness, while MRE adds functional information such as mural edema, best appreciated on T2-weighted sequences. These findings align with Siddiki et al. (2009), who reported strong inter-modality agreement in detecting bowel wall thickening and its correlation with histological inflammation [19]. For mural stratification, agreement was observed in 74.19% of true-positive cases. This feature, characterized by layered bowel wall enhancement, suggests active mucosal and submucosal inflammation. Although CTE’s post-contrast enhancement pattern often provides clear stratification, MRE can demonstrate a similar layered appearance using gadolinium-enhanced T1-weighted sequences. Solem et al. (2008) reported that mural stratification on CT is a reliable indicator of active disease, yet MRE offers comparable visualization without radiation exposure [20].

Skip lesions, a classic marker of CD, were identified with 66.67% agreement between modalities (χ² = 0.0, p = 0.9901). These discontinuous inflammatory segments are well appreciated on both MRE and CTE, though MRE’s multiplanar capabilities offer better visualization of noncontiguous involvement and subtle mural transitions. Oto et al. (2011) similarly demonstrated MRE’s superiority in delineating skip lesions, particularly in pediatric and young adult cohorts [21]. For luminal stenosis, agreement was found in 63.64% of positive cases (χ² = 0.19, p = 0.6589). Luminal narrowing may represent either acute inflammatory edema or chronic fibrotic stricture. CTE’s faster imaging and clearer anatomic delineation make it advantageous in identifying fixed stenoses. Nevertheless, MRE’s combination of contrast-enhanced and diffusion sequences can help differentiate fibrotic from active inflammatory narrowing, an essential distinction for therapeutic planning. These findings are in line with Gourtsoyiannis et al. (2006), who emphasized CTE’s role in accurately detecting and grading bowel stenosis [22].

The creeping fat sign, indicative of transmural inflammation and mesenteric fat hypertrophy, showed agreement in 66.67% of cases (χ² = 0.27, p = 0.6044). MRE is particularly suited for visualizing this feature due to its superior soft-tissue contrast, which allows clear differentiation between mesenteric fat and inflamed bowel loops. Rimola et al. (2011) identified creeping fat as a strong radiological marker of CD and a correlate of disease chronicity [23]. Taken together, these findings underscore that both CTE and MRE exhibit comparable diagnostic performance in most parameters, with MRE demonstrating incremental benefits in mural characterization, soft-tissue contrast, and radiation-free imaging. CTE remains advantageous in acute settings requiring rapid assessment or when evaluating complications such as perforation or abscess.

Limitations of this study should be acknowledged. The sample size (n=35) was relatively small, which may limit statistical power and generalizability. The use of a convenience sampling technique could introduce selection bias. Furthermore, although both imaging modalities were performed on the same day to minimize temporal variation, inflammatory activity may fluctuate within short intervals. The study was conducted in a single tertiary care center, and thus, the results may not reflect variability across different imaging systems or institutional protocols. Motion artifacts during MRI, despite the use of antispasmodic agents, occasionally affected image clarity. Additionally, histopathological correlation was not systematically obtained for all cases, which could have strengthened diagnostic validation.

Despite these limitations, this comparative analysis offers meaningful insights into the complementary roles of CTE and MRE in IBD evaluation. The study reinforces MRE’s value as a noninvasive, radiation-free alternative suitable for serial monitoring, particularly in younger patients and those requiring frequent imaging. Meanwhile, CTE remains a pragmatic choice for rapid assessment in acute or resource-limited scenarios. Clinical implications of this research are significant. The findings advocate for a tailored imaging approach, reserving CTE for acute or emergency cases and utilizing MRE for long-term monitoring and disease activity assessment. Integrating both modalities strategically can optimize diagnostic yield and improve patient safety. Furthermore, establishing standardized imaging protocols and radiologic scoring systems across centers may enhance diagnostic consistency and support evidence-based clinical decision-making.

## Conclusions

This study reinforces that both MRE and CTE are valuable tools in the imaging of IBDs. While CTE is currently the standard, particularly for initial evaluation, MRE demonstrates several advantages, including the absence of radiation and improved tissue characterization. In conclusion, while both MRE and CTE demonstrate high diagnostic accuracy in inflammatory bowel disease, MRE offers superior soft-tissue characterization and functional assessment without radiation, making it the preferred modality for long-term disease surveillance. Future multicentric studies with larger cohorts and histopathological correlation are warranted to validate these findings and refine imaging algorithms for optimal IBD management.
